# Emerging Therapeutic Enhancement Enabling Health Technologies and Their Discourses: What Is Discussed within the Health Domain?

**DOI:** 10.3390/healthcare1010020

**Published:** 2013-07-25

**Authors:** Gregor Wolbring, Lucy Diep, Sophya Yumakulov, Natalie Ball, Verlyn Leopatra, Dean Yergens

**Affiliations:** 1 Department of Community Health Sciences, Specialization in Community Rehabilitation and Disability Studies, University of Calgary, Calgary, AB T2N4N1, Canada; E-Mail: ldiep@ucalgary.ca; 2 Faculty of Medicine, University of Calgary, Calgary, AB T2N4N1, Canada; E-Mails: sophya.yumakulov@gmail.com (S.Y.); neball@ucalgary.ca (N.B.); vgsleopa@ucalgary.ca (V.L.); dyergens@ucalgary.ca (D.Y.)

**Keywords:** health consumer, social robotics, brain machine interface, brain computer interface, neuroenhancement, cognitive enhancement, human enhancement, healthcare, healthcare policy, healthcare ethics, health care ethics, emerging therapeutics, therapeutic enhancement, disabled people, people with disabilities, assessment

## Abstract

So far, the very meaning of health and therefore, treatment and rehabilitation is benchmarked to the normal or species-typical body. We expect certain abilities in members of a species; we expect humans to walk but not to fly, but a bird we expect to fly. However, increasingly therapeutic interventions have the potential to give recipients beyond species-typical body related abilities (therapeutic enhancements, TE). We believe that the perfect storm of TE, the shift in ability expectations toward beyond species-typical body abilities, and the increasing desire of health consumers to shape the health system will increasingly influence various aspects of health care practice, policy, and scholarship. We employed qualitative and quantitative methods to investigate among others how human enhancement, neuro/cognitive enhancement, brain machine interfaces, and social robot discourses cover (a) healthcare, healthcare policy, and healthcare ethics, (b) disability and (c) health consumers and how visible various assessment fields are within Neuro/Cogno/Human enhancement and within the BMI and social robotics discourse. We found that health care, as such, is little discussed, as are health care policy and ethics; that the term consumers (but not health consumers) is used; that technology, impact and needs assessment is absent; and that the imagery of disabled people is primarily a medical one. We submit that now, at this early stage, is the time to gain a good understanding of what drives the push for the enhancement agenda and enhancement-enabling devices, and the dynamics around acceptance and diffusion of therapeutic enhancements.

## 1. Introduction

Health and health care technologies have constantly impacted health and health care and are seen in need of policies to govern them [1,2,3,4,5,6,7,8,9]. Health and health care technology, and the policies around them, so far, are based on a meaning of health (and therefore health care) that is benchmarked to the normal or species-typical body. However an increasing amount of therapeutic interventions have the potential to move beyond their restorative purpose and give recipients beyond species-typical body abilities (therapeutic enhancement, TE). To clarify, we do not understand TE to mean enhancement from a sub-species typical level to the species-typical level (restoration) nor incremental changes to the abilities of the body within the species-typical variation range. We are interested in the cases where TE leads to whole new abilities not possible for humans. The bionic cheetah legs of Paralympic athlete Oscar Pistorius [10,11,12,13] are just one example of therapeutic devices with TE potential (they are not there yet, but might enable beyond species-typical abilities down the road). Other envisioned therapeutic devices that could lead to TE are artificial and cyborg organs [14], artificial hippocampus [15] (an implanted chip that acts as a repository for the memory we generate), brain machine/brain computer interfaces [16], sub-vocal speech devices [17], social robots [18], sensors embedded in health technologies [19], and drugs and devices used for neuro and cognitive enhancement [20,21,22]. Cochlear implants or hearing aids, might eventually lead to beyond species-typical hearing [23].

We submit real TEs are enabling a paradigm shift as to the meaning of health and body ability expectations [24] where we see the move toward a sentiment that links being healthy to having obtained more than species-typical abilities, abolishing, as a consequence, the species-typical framework of reference evident in medicine up to today [9,25,26]. Another consequence already evident is the appearance of an enhancement form of ableism (ability one sees as essential) that expects beyond species-typical abilities of humans [27].

TEs will have various impacts on the health care system and the policies around them. Both potential users and producers might lobby, among others, for the field of enhancement medicine and changes in health insurance plans and healthcare delivery [25]. New health practitioner jobs might be created such as “body engineers” (people who design bodies around enhanced abilities) and the job description of other health practitioners might change; for example speech therapy might not be about training a person verbal speech any more but about how to transmit thought through other devices.

However, so far mostly non-therapeutic performance enhancements are debated by policy makers, scientists and laypeople with various ethicists arguing for the legalization of various forms of enhancements [27]. Therapeutic enhancements as such are rarely questioned and their impact is rarely discussed [27]. A 2009 “Human Performance Study”, written for the Directorate General for internal policies, Policy Dept. A: Economic and Scientific Policy Science and Technology Options Assessments of the European Parliament, concluded that human enhancements would put a strain on social solidarity and on healthcare systems and might impact health budgets [28]. The report noted that no platform exists in Europe for monitoring and discussing human enhancement issues and for bridging the gap between the needs and the concerns of the broader public and the practitioners and experts [28]. Therefore our study investigated the following research question: how is healthcare and related areas such as health care policy, ethics, public health, and evidence-based medicine discussed around TE?

Various types of assessment discourses such as health technology assessment (HTA) [29,30,31,32,33,34,35], health impact assessment (HIA) [36,37,38,39], health needs assessment [33,40,41], social impact assessment (SIA) [42,43,44], parliamentary technology assessment (PTA) [45,46,47], and participatory technology assessment [48,49] exist with the mandate to investigate health technologies. Our study looked at which forms of assessment covered various therapeutic enhancement discourses and products.

People increasingly demand that they can shape scientific and technological research and development [48,50,51,52,53,54,55] and the same demands are also apparent within the health care intervention area [52,56,57,58,59]. As it is stated by Ableson, Giacomini, Lehoux, and Gauvin (2007), “a growing consumerist orientation to health care policy routinely draws health system “users” into consultations, evaluations and decision-making processes about health technology by health planners and managers” [60]. Therefore our study investigated the visibility of TE discourses and products in the health consumer discourse and whether the different TE discourses talk about health consumerism.

Finally, disabled people are at the forefront of TE. It is anticipated by disabled people [23] and others [61] that non-disabled people will desire the use of many therapeutic enhancements, especially if they are removable and non-invasive, and that therapeutics that enable enhancements beyond the species-typical might be obtained and marketed within and outside of the health system. Therefore our study looked at the imagery and involvement of disabled people in the discourse around TE. 

## 2. Experimental Section

### 2.1. Theoretical Framework

Disability Studies is one lens through which we investigated the literature. Disability Studies is an interdisciplinary/multidisciplinary academic discipline that investigates the situation disabled people face, which includes the investigation of their imagery and involvement in existing discourses [62,63].

A participatory framework [56,57,58,64,65,66,67,68,69,70,71,72] was used to look at the narrative around the consumer in general and health consumer in particular. A technology assessment lens was used to look at the assessment of TE including terms such as evidence and evidence-based, as evidence is needed for assessment. Using a health care governance lens, we focused on how health care was mentioned in the documents, what guidance for health care governance was evident within the narrative of health policy and health ethics.

### 2.2. Data Sources

#### 2.2.1. Brain Machine Interface (BMI)

The following databases were searched in July 2012 for BMI literature through the University of Calgary: ScienceDirect, Scopus, OVID (All), EBSCO (All), Web of Science, and JSTOR. From this search, 1058 articles were found and imported into Knowledge Share (KSv2) version 2.1.3 [73]. This tool was used to systematically review the literature by process of inclusion/exclusion based on the following criteria—include: full PDF available articles, written in the English language; exclude: books, conference announcements, and purely technical articles. With an abstracts Kappa score (the primary and secondary researcher agreement) of 0.99, 6.7% (n = 71) of the articles fulfilled the criteria and were included for review. The coding process was performed using the qualitative data analysis and research software, ATLAS.ti^©^ version 7.0.75. The literature underwent separate analysis by two researchers in Atlas.ti 7 to increase the reliability of our findings.

#### 2.2.2. Social Robotics

Academic articles covering social robotics were searched in May 2012 and imported into Knowledge Share ver. 2.1.3 (KSv2) [73]. We searched the following databases (provided by the University of Calgary): Science Direct, Compendex, IEEE, Communication Abstracts, Scopus, OVID (All), EBSCO (All), Academic One File, Web of Science, and JSTOR. 489 articles were imported into Knowledge Share [73], and using the same inclusion/exclusion criteria as for the BMI literature, we included n = 171 articles in the review (34.9%). The Kappa factor (reviewer agreement) was 0.88. Articles were coded using Atlas.ti 7 qualitative data analysis software. The literature underwent separate analysis by two researchers in Atlas.ti 7 to increase the reliability of our findings.

#### 2.2.3. Neuroenhancement, Cognitive Enhancement, Human Enhancement

This literature was collect in June 2012 from JSTOR, ScienceDirect, PubMed, EBSCO (All), Web of Science and Scopus (Elsevier) and imported into KnowledgeShare [73]. These articles were found using the terms “neuroenhancement”, or “cognitive enhancement”, or “human enhancement”. Inclusion criteria were: full-text English, and addressing neuro/cognitive/human enhancements *within humans* in a non-rehabilitative fashion; that is, the enhancements that are being used to increase capabilities above what is considered to be “normal”. Articles were reviewed for inclusion separately by two researchers. Using the initial keyword search, we found 1,022 articles for cognitive enhancement, 361 for neuroenhancement, and 373 for human enhancement. A Kappa factor of 0.79 for the articles found using the phrase “cognitive enhancement”, 0.90 for articles with the term “neuroenhancement” and 0.92 for articles with the term “human enhancement” were obtained between the two researchers. Any disagreements were addressed individually until a consensus could be reached for the article in question between the two researchers. Once inclusion and exclusion criteria were applied and duplicates were deleted, 82 articles (8.0%) for cognitive enhancement, 61 (16.8%) for neuroenhancement, and 40 (10.7%) for human enhancement underwent content analysis using Atlas.ti software. Articles then underwent thematic analysis, wherein the texts were coded for content related to the research questions. The literature underwent separate analysis by two researchers in Atlas.ti 7 to increase the reliability of our findings.

### 2.3. Coding

We used ATLAS.ti^©^, a qualitative data analysis software (CAQDAS) [74,75], for generating qualitative and quantitative data. ATLAS.ti^©^ allows the researcher to analyze imported sources of data that can consists of PDF, Word, html, audio, and video files. After all sources were imported into ATLAS.ti^©^ we performed hermeneutical keyword coding. We employed various coding strategies, one being a deductive strategy where we used a set of predetermined terms fitting the coding analytical frameworks employed and the research questions. This list was a starting point but by no means the endpoint. The list was used as a starting point for using the auto-code function of ATLAS.ti^©^, which allows one to search all the documents of each research sub-project for a given word. For example all our sources were searched for the terms “impairment” and “disab*” (catching disabled, disability, disabilities) allowing us to analyze the imagery of disabled people in the documents of the different discourses we investigated.

We also employed an inductive and iterative coding strategy, in which articles were read and when a theme was identified we used the free coding option to generate new themes. Free coded themes are added to the coding list.

For any given source at least two authors performed the coding to increase reliability, and differences were resolved during our discussions.

Following coding we used ATLAS.ti^©^ to generate the frequency of certain themes (quantitative data) and to describe in a narrative way the content linked to the themes (qualitative data).

### 2.4. Limitation

In the case of BMI, social robotics, and neuro/cognitive enhancements, other terms exist that also could lead to applicable results; namely, we could have excluded terms such as human machine interface into the BMI analysis and we could have used terms such as companion robots to add to the social robotics literature.

## 3. Results and Discussion

### 3.1. Quantitative Data of the Themes Covered in Section 3

#### Discussion

The results shown in Table 1 highlight that certain terms relevant to the healthcare discourse are hardly covered in the literature we investigated, and some are covered unevenly between the different discourses. To highlight a few areas: although 85.9% of the BMI literature mentioned the term “patient”, thereby indicating a medical focus of the articles, healthcare only showed up in 9.8%, and healthcare ethics, healthcare policy, health consumer, health ethics, and health policy did not show up at all. Given that patients obtain their treatment through the health care system, we submit it being problematic that the healthcare system is not a topic of engagement. This problem, outlined for BMI articles, also exists for the social robotics and human enhancement and neuro/cogno enhancement literature as Table 1 shows. Another troubling issue exists with the low frequency of the term “evidence based”, given that health care interventions should be provided based on solid evidence. Finally, technology assessment is seen as a framework that can ascertain the usefulness of a given technology and its impact; however, Table 1 indicates that technology assessment is not an issue as of yet. Many of the issues evident from the results in Table 1 are expanded upon in different sections using qualitative data we obtained to deepen the points.

**Table 1:** Quantitative data of the themes covered in Section 3.

Health care discourse relevant Terms	Human enhancement (n = 40 article = 100%)	Neuro- enhancement (n = 61 article = 100%)	Cognitive enhancement (n = 82 article = 100%)	Brain machine interface (n = 71 article = 100%)	Social robotics (n = 171 article = 100%)
	Count (%)	Count (%)	Count (%)	Count (%)	Count (%)
Assessment	40.0	42.6	58.5	33.8	29.8
Consumer	20.0	16.4	8.5	12.7	4.7
Disab	27.5	14.8	15.9	38.0	21.1
Ethics	90.0	90.2	75.6	19.7	18.1
Evidence	70.0	63.9	24.4	62.0	41.5
Evidence based	0.0	11.5	3.7	2.8	0.0
Global	27.5	78.7	24.4	25.4	2.9
Global Health	2.5	3.3	1.2	0.0	0.0
Health	87.5	90.2	87.8	64.8	48.5
Health care or Healthcare	25.0	26.2	13.4	9.8	8.7
Healthcare ethics	0.0	0.0	0.0	0.0	0.0
Healthcare policy	0.0	0.0	0.0	0.0	0.0
Health consumer	2.5	3.3	0.0	0.0	0.0
Health ethics	5.0	0.0	2.4	0.0	2.3
Health policy	5.0	0.0	2.4	0.0	14.6
Impairment	17.5	29.5	23.2	32.4	10.5
Patient	27.5	57.4	63.4	85.9	28.7
Policy	67.5	88.5	47.6	11.3	21.6
Public Health	2.5	14.8	15.9	2.8	0.0
Technology Assessment	5	3.3	2.4	1.4	0.6

### 3.2. Qualitative Data: How Are Health Care and Some Linked Issues Discussed?

#### 3.2.1. Neuroenhancement

##### 3.2.1.1. Health Care

The issue of health care was mentioned in n = 16 articles. One article linked the “increasing demand for a competitive ‘edge’ to maximize skill-driven, technologically nested social incentives in which cognitive abilities like memory, attention, alertness, and mood are judged to ‘make a difference’ ” to an increase in value and public acceptance which in turn is seen to lead to a “progressive desire for ‘neuroenhancement’ among the young” [76]. This dynamics is seen to require an exchange of views “among researchers, healthcare practitioners, ethicists, the public, and those who inﬂuence and enact guidelines and policies” [76]. Another article highlights that various people, visible in the neuroenhancement discourse, have labeled neuroenhancement as legitimate health care [77]; another recommended that “health care systems and pharmacies should institute systems to monitor the quantity and location of requests for neuroenhancers to avoid abuse and monitor total dosage” [78].

##### 3.2.1.2. Disability

Searching the articles for the term “disab*” which covers disability, disabled, and disabilities led to n = 9 articles, while impairment led to n = 18 articles. All but one article used these two terms to simply highlight a medical “condition”, a deviance from “normal” abilities and bodies.

Only one article, although still thinking in terms of deviance, covered a social dynamic namely the possibility that neuroenhancement may lead to a change in the concept of “normal” and lower tolerance of cognitive and other differences and disabilities seen as deficient [78].

##### 3.2.1.3. Health Care Policy and Ethics

The articles that looked at neuroenhancement (n = 62) in general had various policy concerns that we see to fall also under health policy issues. With respect to health policies for neuroenhancement, it is mentioned that policies have to be broader than individual-level use [79] and have to consider the impact on identity of the consumer [80] as well as the wider social and personal functioning/values affected by neuroenhancement [81]. It is suggested that access should favour the disadvantaged and make it more difficult for the advantaged [82]. It is believed that policy-makers must make accurate, comprehensive knowledge about neuroenhancements available to the general public [83] in order for the public to make up their minds [84]. Singh and Kelleher, for example, make a case that more research and evidence, particular in terms of prevalence, safety and efficacy is needed [78]. They believe that neuroenhancers should be monitored by Pharmacies and health care facilities as a means to mitigate abuse. Self-perception (before and after) and social coercion (before) should be measured to limit it as a motivator for enhancing [78]. Clinical and social effect should be documented and researched [78]. Neurologists should have the right to refuse prescribing neuro-enhancements [85] and prescription of neuroenhancing drugs should be strictly based on harm/benefit ratio [86].

In terms of wider policy issues that could stretch beyond health policy, various concerns are raised. Some perceive policies of banning neuroenhancements as coercive and paternalistic [87] and called neuro-enhancements policy decisions premature [88] because policy may have poor basis because of inflationary claims about neuroenhancement in the media [20,89]. Singh and Kelleher suggest that schools/teachers should not be allowed to attempt to coerce parents into giving their children neuroenhancers and should receive ethical training, and that use of neuroenhancers with abuse potential should be limited [78].

#### 3.2.2. Cognitive Enhancement

##### 3.2.2.1. Health Care

Twelve articles mentioned the term health care whereby one article made the argument that new enhancement options not be banned but that their appearance is linked to inequality-reducing measures, which the authors suggest could be “making the enhancement available via public health care at an affordable price” [90]. Another article concluded that “multidisciplinary, empirical research on attitudes toward cognitive enhancement is essential to a more informed ethical debate that will shape the responses of health care professionals, policymakers, and the general public to the use of stimulants and other pharmaceutical drugs for cognitive enhancement” [91]. Outram *et al*. outlined their effort to develop public health approaches to cognitive enhancement, which depend on much better quality and quantity of data [92].

##### 3.2.2.2. Disability

Twelve articles mentioned disability, all but two with medical connotation, and the ones with no medical connotation were using disabled people to sell enhancement. One article stated: “Anita Silvers, the prominent San Francisco State University-based bioethicist and disability-rights advocate, writes that such modifications are a basic human right and the very ‘essence of freedom’ ” [93].

Another one gave voice to the paraplegic Journalist John Hockenberry who supports various assistive technologies some of which are classified in the article as cognitive enhancements (such as BMI) [94]. Three articles highlight the benefits of enhancements to disabled people. One article quoted Cyberkinetics company (BMI producers) spokesperson, “we can help people who are disabled become super able in a new sense” [94]; a second one ascertained that “technologies will continue to be developed and refined, offering disabled users greater access and control over their lives” [95] and a third mused that “germ-line enhancement might lead to better treatment of people with disabilities, because a general demystification of the genetic contributions to human traits could make it clearer that people with disabilities are not to blame for their disabilities and a decreased incidence of some disabilities could lead to more assistance being available for the remaining affected people to enable them to live full, unrestricted lives through various technological and social supports” [96].

##### 3.2.2.3. Health Care Policy and Ethics

As to policies mentioned in the cognitive enhancement discourse (n = 82 articles), it is believed that if policies are made it has to be before the technologies are fully developed [93]. These policies transcend the health policy level and reach into broader societal issues. It is mentioned that in order to maximise autonomy, direct or indirect coercion has to be limited [93]. It is recognized that policy-makers need to acknowledge that cognitive enhancements are not just individually valued, but can contribute to greater social goals and values and it is suggested that policy should be based off of risk, suggesting that if the risk is not more than other activities done for leisure, then it could be made available with the appropriate warnings [97]. Some suggest that wider social implications have to be considered [98], that it should be children’s and not the parents’ decision to take cognitive enhancers and that youth therefore have to be involved in the policy process [99]. Various thoughts emerged around the topic of evidence. These included the epidemiological evidence approach, risk/benefit approach, and historical/social context approach of policy development [92]. Various suggestions were made focusing more directly on the health aspect of cognitive enhancement. High-quality evidence on the use of stimulants for enhancement in order to formulate policy is seen as needed [20], but at the same time it is feared that sound evidence is not accessible and that research is needed on the average person’s attitude toward cognitive enhancement [100]. Some think about different risk benefit levels [101] and suggest that if benefits outweigh risks, the government should consider subsidizing the cost of cognitive enhancements for individuals [101]. One study suggests that we should not cognitively enhance beyond the normal because it could endanger our survival [102].

#### 3.2.3. Human Enhancement

##### 3.2.3.1. Health Care

Eleven articles mentioned the term health care. Notably, one concluded that it is important to make a distinction between enhancement and therapy because it helps “to deﬁne the proper goals of medicine and biomedical research” and it helps “to determine the limits of the health care payment system (what should be reimbursed?)” [103]. One article contended that Human growth hormone treatments for the elderly could dramatically affect health care [104]. Another article gave voice to the argument against enhancements that “result in serious health risks” and “take resources away from basic health care provisions” [105].

##### 3.2.3.2. Disability

As to the topic of disability, 10 articles mentioned disability and ten the term impairment, all but two [106,107] with medical connotation.

##### 3.2.3.3. Health Care Policy and Ethics

As to the issues mentioned in the n = 40 articles covering human enhancement, 5 mentioned health policy. It is felt that ordinary citizens should be involved in the debate and policy making [108]; that policymakers need to acknowledge moral and scientific assumptions from genetic determinists, and should make the distinction between what science *can* and *ought* to do [109]; that traditional medical ethics can’t capture all of the relevant variables to do with human enhancement and that policymakers need to consider wider social implications [110]; that biogerontology may be a useful model to draw from when formulating human enhancement policy [111]; and that knowledge, other than scientific evidence, may be appropriate when making policy [112].

#### 3.2.4. Brain Machine Interface (BMI)

Of the 1,058 BMI articles, 987 discussed purely technical issues such as the merit of invasive versus non-invasive approaches to BMI, the different signals used to measure brain activity and the effectiveness (or ineffectiveness) of the different approaches. On the application side, articles focused mainly on medical/therapeutic needs—patients with disabling conditions such as: Amyotrophic Lateral Sclerosis (ALS), Parkinson’s disease, spinal cord injuries, and Cerebral Palsy. Discussion about human enhancement was, if mentioned, linked to application in the military, space and gaming/entertainment and not linked to therapeutic interventions. However, if enhancements are possible within military applications it seems reasonable to assume that enhancements could also be obtained by people using BMI as therapeutics. Only 6.7% (n = 71) article covered non-technical aspects. We submit that this is rather underwhelming coverage.

##### 3.2.4.1. Health Care

Of these, 9.8% mentioned healthcare, although there was limited in-depth discussion about the implications of BMI technology for healthcare systems and healthcare in general. Some articles mentioned that health care professionals and researchers have an interest in BMI [113,114,115], while others pointed out that BMI technologies for health applications most often come as “hand-me-downs” from other industries [116]. Some healthcare challenges were mentioned, such as the high expectations of patients and their limited understanding of the risks and benefits of BMI [117], and resistance to technological eldercare, even though this is predicted to reduce healthcare costs of eldercare [118]. In general, healthcare or health systems considerations were not the primary focus of these articles, but were mentioned only in passing.

##### 3.2.4.2. Disability

Nearly 40% of articles discuss people with disabilities as the main target for BMI intervention; most conceptualize people with disabilities as patients in need of a means to improve communication [119,120,121,122], and motor skills [121,123,124,125]. The common theme among these articles is the desire to give physically disabled people a “quasi-normal life” [126].

##### 3.2.4.3. Health Care Policy and Ethics

As to health policy, only one article looked at health policy/health care policy [127]. The following is covered in this article as policy challenges:

(a) “to ensure adequate safeguards, regulation and transparency to support a society of increasingly cognitively resourceful individuals, and also to moderate unrealistic expectations of infallibility”
(b) discussed how public policy and regulations can:
  - contribute to inequality by “driving up costs, limiting access and creating black markets”
  - reduce inequality by “supporting broad development, competition, public understanding and perhaps subsidized access for disadvantaged groups”
(c) discussed the obstacles to develop and use cognitive enhancements:
  - licensing drugs and medical treatments: the system is geared toward dealing with traditional medicine for treatment, cure, prevention, diagnoses, or alleviation of disease. Medicine to enhance the human body is not within the framework. Access to enhancers then become dependent on individuals being able to find a physician “open-minded” enough to provide a prescription, therefore creating inequity because individuals with “higher social capital and good information” will get access, excluding others.
  - change in patient-physician relationship—patients are becoming more informed and involved in their health, being aware of treatments and options for their condition (information accessed from Internet services). Suggests the need for regulatory change to inform individuals from risks, ensuring they understand the risks before undergoing enhancement procedures.

Possible options of how to deal with challenges indicated were:

- establish baseline level of acceptable risk;
- develop enhancement licenses to ensure informed consent is received and enables better monitoring; barrier for individuals with low cognitive capacity;
- there is a need for more public funding research to reflect potential personal and social benefits of cognitive enhancements;
- cultural challenge of de-stigmatizing enhancers; perception is that taking medicine is a “regrettable activity” and the use of non-therapeutic medicine raises suspicion and possible misuse;
- testing of cognitive enhancements need to take place in natural environments rather than in labs;
- would access to enhancements be regarded as a human right? Banning enhancements would result in black market sales, limiting social benefits;
- legalizing enhancements would result in affordable and safe enhancements.

This is obviously not sufficient given that one of the main areas of BMI’s is envisioned to be therapeutic.

- ethics issues often cover policy issues that also effect health policy issues. Of the 1,058 articles covering brain machines 1.9% (n = 21) of articles covered ethics issues. The following ethical issues with health policy implications around BMI are raised in the literature: there is currently no legislation put in place to “safeguard the informed consent of the patients before a BMI is used in therapy” resulting in issues around the protection of patient privacy [128];
- concerns are raised with regards informed consent from minors and incompetent individuals—who should be making the decision to provide or not provide consent for TE? [127];
- the need for protocols with implementing BMI application for humans “in terms of acceptable risk to subject” [127];
- research reliability comes into question with small effect size. This runs unforeseen risks upon exposure to the greater population [127];
- risk of human dependency on “outside technology, infrastructure, or drugs” which could result in experiences of withdrawal or impairments [127];
- ethical and quality of life issues around BMI research and application [129].

#### 3.2.5. Social Robotics

##### 3.2.5.1. Health Care

The coverage of healthcare within social robotics literature is discussed elsewhere [130]. In general, social robots are currently in use, and envisioned to be used, in a variety of areas. These include rehabilitation therapy, eldercare, and autism. Of the articles obtained in our database searches only seven covered health care beyond a single sentence [131,132,133,134,135,136,137].

##### 3.2.5.2. Disability

The imagery of disability lens within social robotics literature is discussed elsewhere [130]. In general many robotics applications are related to people with disabilities and the discourse around disabled people mainly reflects a medicalized, “fixing” framework, and deals with disabled people within a patient, health care consumer framework. 

##### 3.2.5.3. Health Care Policy and Ethics

Within the social robotics literature, 34.9% (n = 171) of articles cover issues that go beyond purely technical issues and therefore fare better than the BMI literature (6.7%). However, this does not translate into more coverage of health policy; out of 48 health-related articles, only 12 (25%) mention policy and only one article mentioned the term “health policy” and none the term “health care policy”. Coverage of policy-related issues was limited, usually only mentioned in passing, and seldom related to healthcare. One study surveyed members of the public, experts, and policy makers about the acceptability of autonomous robots; however, the kind of policy makers involved was not specified, and there was no discussion of implications for healthcare policy [138]. Another article mentioned the importance of policy in developing safety standards [139], and one article warned that a lack of consensus on ethical issues around robotics has led to a policy vacuum [140]. Although policy and law is mentioned in regards to robot-governing policies and consortia, health policy or policies discussion for health applications are absent. Only one article [141] spoke about the importance of consumer involvement in policies governing robot use in eldercare. Overall, health policy discussions are not present in social robotics literature despite the fact that research continues to focus on healthcare applications.

As to ethical issues, numerous articles covered ethical issues related to robots extensively. As to the topics covered, they included the ethics of how to treat the robot [142], how people decide what are ethical applications of robots [138], the issue of robot sociability problems [143], how to create well-behaving artiﬁcial agents [144], about the socio-ethical resistance of workers [139], human robot interactions [145], socially situated perspective on the ethics of robot design [146], and ethics of robot companions [147]. However most of the ethics discourse is not linked to the health care field. One exception is the article by Sparrow and Sparrow who investigated the application of robots in eldercare and concluded “ethical uses of robots in the aged-care sector are far fewer than ﬁrst appears. More controversially, we believe that it is not only misguided, but actually unethical, to attempt to substitute robot simulacra for genuine social interaction” [141].

#### 3.2.6. Discussion

Our data show that between 48–90% of the articles cover health as an issue and the mentioning of the term patient present between 28–80%. However, health care is mentioned much less. Furthermore, although certain governance topics are visible within the documents such as policy and global and ethics, their health and health care counterparts are much less of an issue. Although there is an acknowledgment of the global aspects of the topics we studied, global health was not mentioned. Ethics and policy were terms mentioned but health or health care policy and ethics were not visible. Although some policy issues are raised, they are by no means extensive. Furthermore they are often raised by ethicists but not by health policy scholars. We submit that all the science and technology products and discourses covered in this paper will impact the delivery of healthcare and the quality indicators of health, healthcare and health systems such as the ones used in Canada [148,149]. They already raise various health policy issues today [9,25,26] such as enabling a changing understanding of health itself [9], although health-related terms such as disability adjusted life years, burden of disease and social determinants of health [25] were not visible in the articles we investigated (data not shown). A 2006 Association for the Advancement of Science workshop [150] looked into the dynamic of human enhancement and concluded that the following ability desires are the main drivers for human enhancements:

(1) to keep one’s local and global competitive advantage;
(2) to live securely;
(3) to maintain one’s quality of life and one’s consumer life-style.

We submit that these drivers must have an impact on health and healthcare, especially at the public health level. Proponents of public and individual good exhibit different ability preferences and their discourses are part of a bigger discourse around justice and health [151,152,153,154,155,156,157,158]. Different theories of distributive justice [159], and different substantive principles of justice [160], are seen to offer different priority setting regimes for health services. Some try to bring the often conflicting theories of justice together. Expectations and desires are discussed within the justice and health arena [161,162,163] and various models are deployed to evaluate the views of the public on how to prioritize healthcare [164,165,166,167] and what health technologies to use [168]. Depending on which abilities one accepts as essential, one could invoke different theories of justice to generate health policies around TE. 

Given that there might be some distributive justice issues [169] we submit the topic is of interest to public health, which is about the impact at the population level. However, for the most part public health is an angle invisible in our literature. Outram and others outlined the emerging development of a public health framework for cognitive enhancements, as they perceive it [92]; they performed a content analysis of two reports, one being the British Medical Association Medical Ethics Committee report entitled “Boosting your brainpower: ethical aspects of cognitive enhancement” and the other being Commission de l'éthique de la Science et de la technologie du Québec report on “Psychotropic drugs and expanded uses: an ethical perspective” [92]. However one of their main conclusions was that it is not quite clear yet whether cognitive enhancement has positive or negative consequences for public health or whether it is of no significance for public health at all. Others predicted grave consequences for public health [170]. Bostrom makes the case that it might be best if cognitive enhancements are made available through public health care [90], which McNamee questions will happen [96]. It is suggested that regulating cognitive enhancing drugs could be a useful public health measure [99]. It is recognized that attitudes towards nonmedical use of stimulants is to differ based on whether it is framed as a public health problem or a lifestyle choice [91]. The current lack of health narrative might be an indication that we are moving toward the lifestyle option.

On the other hand the social group of disabled people were dealt with nearly exclusively within a medical/health framework ignoring the social aspect of the disablement of disabled people. 

### 3.3. What Does the Health Consumer Discourse Cover?

In this section we report content and visibility around the term consumers within Neuro/Cogno/Human enhancement and within the BMI and social robotics discourses. 

#### 3.3.1. Neuroenhancement

Although health-consumers are only mentioned one time, consumers are covered in n = 10 articles. Neuroenhancement is seen as having the potential to cause self-alienation of consumers and “impede their identiﬁcation with new traits, rendering them nonautonomous” [80]. Neuroenhancements are linked to commercial interests as demonstrated by Direct to Consumer Advertising (DCTA) [171]; some see problems with DCTA [172] and others compare neuroenhancement with energy drinks stating that if they are proven safe they should be allowed as over the counter and the marketing should be allowed. As long as there is not enough data on safety and efficacy they should not be marketed as lifestyle products [78]. One article contends that it will be a challenge to protect consumers, citing the problems with “ ‘natural’ forms of enhancement in the form of nutraceuticals, functional foods and dietary supplements” [173]. One paper states that neuroenhancements are already accepted by consumers and that this is a market for companies and “for physicians who might enter the potentially lucrative specialty of so-called cosmetic neurology” [174]. In addition, one highlights “ ‘neuromarketing’ as the latest tool in accessing consumer desires and enhancing sales” [175].

#### 3.3.2. Cognitive Enhancement

Consumers are covered in n = 7 documents whereas the term “health consumer” is not mentioned once. As to how consumers are mentioned, one article feels that neuroenhancements should be regulated for safety issues as other medical goods and services, and that consumers should be informed, but neuroenhancements are not seen worthy of special precaution as they see no documented evidence of higher risk [93]. One article sees consumers as being players in the debate [176]. One article thematizes the “pharmaceuticalization of daily life as consumers come to see such substances as “magic bullets” to resolve their everyday problems” [177] and that whether something is marketed as medicine or consumer products has implications for how they are regulated and perception as to health benefits and safety and level of acceptability [177]. One article writes about how health problems are redeﬁned and reconstructed as having a medical/pharmaceutical solution, how new social identities are created and how patient/consumer groups are mobilized around drugs [177]. The same article highlights that “over the past decade, instead of developing pharmaceuticals as medicines for disease and disorder, the emphasis has been on lifestyle in the production, marketing and consumption of pharmaceutical products” [177]. One article links the politics of identity and the self to the rise of consumerism and restates a question by Elliot of whether this is “an authentic self or one invented by altering the biology (through surgery, medicines, or devices) and imagined through advertising, media, health advisory groups, or other images” [94]. One article states that recipients of neurointerventions “are consumers, making access choices on the basis of desires that can appear trivial, narcissistic, or irrational, shaped not by medical necessity but by the market and consumer culture” [178].

#### 3.3.3. Human Enhancement

Eight articles mention consumers whereas none mention the term health consumer or health care consumer. One article wrote about human Growth Hormone (hGH) and how concerned citizens, consumer activists, and scientists questioned various issues posed by enhancement and cosmetic applications of hGH [104]. One article questions the apathy of physicians against “cosmetic medicine” seeing “it as an inevitable path of the American way. The logic goes like this: If the patient is just a consumer buying a service, well then, caveat emptor; not our problem to worry too much about deals made between consumers and providers” [179]. A 2011 article mentions Haga and Willard providing a framework that helps to understand government public health activities in the areas of emerging scientiﬁc and technological breakthroughs. The framework has ﬁve regulatory dimensions (research issues, legal issues, economic issues, education issues and acceptance and implementation issues) and specific issues such as intellectual property rights, public information, commercialization of retail products, safety, health, consumer choice, trade, and research investment [180]. One article highlights that “ “Normality” is subjective to peer, societal, cultural and/or ethnic standards, dictated by mediatized consumerism, which generates discourse-legitimizing conceptions of an ideal, and promotes the means of attainment” [181] and that “ ‘Consumerist language’ is increasingly used in discussing enhancement interventions” [181].

#### 3.3.4. Brain Machine Interface (BMI)

In terms of consumer perspectives, the BMI literature mainly discusses the public as commercial consumers, rather than health consumers, and as with the limited focus on healthcare issues, consumers are likewise seldom discussed in the context of healthcare. Most studies conceptualize BMI technologies as commercial products rather than health services; they discuss market evaluation of consumer acceptance and feedback [182], and the availability of information about technologies available to aid in consumer decision-making [183]. Only one study mentioned health consumerism, positing that it will be the informed and active health care consumer who will drive the promotion of cognitive enhancements for preventative and enhancement purposes within a medical and health care context [127] whereby the “informed health care consumer” is seen to “insists on exercising choice” [127].

#### 3.3.5. Social Robotics

The term consumer was evident in various articles. One study stated the following: “Studies by the United Nations Economic Commission and International Federation of Robotics forecast a dramatic increase in consumer demand for robots that assist, protect, educate and entertain over the next 20–30 years” [184]. Another study made the claim that “It is becoming accepted, however, that innovation in consumer environments is highly dependent upon factors of socialization that merge utility with symbolic and cultural factors, and that this involves subtle transfers of knowledge from consumers to producers about emerging social trends and preferences” [185]. One study employed a user satisfaction questionnaire to ascertain the satisfaction of user with the consumer product [186]; one study looked at how to change the design in order to increase acceptability by consumers [139]. However, no articles used the term health consumer or health care consumer and indeed the applications mentioned around the term consumer were not mentioning health care applications but focused on domestic robots and toy robots rather than on health care robots.

#### 3.3.6. Discussion

Our analysis reveals a disconnect between the consumer narrative around enhancement discourses and BMI and social robot devices and the health and health care domain. Although the role of consumers as being influential and being influenced are acknowledged, no linkage is made to the health consumer or health consumerism discourse. It is well known that the commodification of the body through body technologies [187] plays a role in health, medicine and care [188,189], cosmetics [190,191,192], and especially the posthuman/cyborg discourse [193,194,195,196,197,198]. Furthermore, given there is increasing evidence that health clients want to be in the driver’s seat with their health interventions [199]; the increased engagement with patient-driven healthcare and research models [200,201]; the movement towards a “quantified self” (where people diagnose themselves) [201,202,203,204]; and the increase in health social networks and the active health technology market that makes consumer personalized medicine [205,206,207,208] possible, we submit it being problematic that the linkage between consumer behaviors and “health consumer” is not more explicitly investigated. 

It is troubling that only one article written by authors based within a pro-enhancement think-tank acknowledged the scenario that health care consumers will drive the uptake of the enhancements using the argument of choice [127]. Indeed, given the ample evidence around how much health consumers are influenced by external factors from advertisement to social factors, from product producer-driven medicalization [209,210,211,212,213,214,215,216] which tries to make the healthy feel bad about themselves, to consumer-driven forms of medicalization where the consumer makes the case that the product is essential for their own health (see e.g., cosmetic surgeries which were first elective and are now often being considered as necessary for the psychological wellbeing [217,218]), we would expect more engagement with health consumers and health consumerism. This shift in the nature of the health client from a passive recipient to an active shaper, and the framing of patients and clients as consumers has broad implications for health systems [219] and policy developments [201,220,221,222,223,224,225,226,227], and we submit that it is worthy to investigate the impact of enhancement within the health care literature. 

### 3.4. Who Does Make the Assessment?

In this section we report how visible various assessment fields are within Neuro/Cogno/Human enhancement and within the BMI and social robotics discourse.

#### 3.4.1. Neuroenhancement

The term assessment showed up n = 132 times in n = 22 articles with many of the incidents not related to the enhancement section of the article. As to the relevant ones, one study suggested the need to assess the consequences to common morality and public acceptance [228]; another indicated the need for studies “aimed at identifying appropriate screening, assessment, and monitoring tools in any medical setting, and we add that integrative care paradigms and methods need to be studies” [76]; and one highlighting that they assessed the motivational factors behind using neuroenhancers [229]. Another stated that the assessment of legal risk is difficult [174], assessment of impact on important values [82] should be performed and the authors question the assessment by others “that psychotropic neuroenhancers are not, in morally relevant ways, different from other forms of enhancements” [82], “A point often raised in discussions regarding enhancement is the common liberal position that the ultimate duty of a physician is to respect the patients’ own assessment of what constitutes proper care” [77]. Risk assessment is mentioned twice. However technology assessment was only mentioned in one article stating the need for technology assessment, and other methods of analysis and reﬂection on the interface between technology and society [230]; impact assessment or needs assessment were not mentioned at all. 

“Evidence-based” as a phrase did show up seven times. One article believed that physicians should not just give neuroenhancement to “patients” because they wish it but evidence has to be there [174]; one paper highlight that “recently a number of experts called for an evidence-based approach to the evaluation of the risks and beneﬁts of cognitive enhancement” [231]; “Such regulation must be evidence-based and a product of active dialogue between scientists, doctors, ethicists, policy-makers and, importantly, the general public” [232]. Another stated that evidence-based approaches were needed [78]. As to the term evidence, it was used n = 181 times. As the evidence relates to neuroenhancements, 12 articles stated that there was a lack of evidence; two stated that there was evidence of increased acceptance and three that there was evidence that neuroenhancements are achievable.

#### 3.4.2. Cognitive Enhancement

As to assessment, the term shows up 138 times with various issues seen in need of being assessed; impact assessment or needs assessment were not mentioned at all. Evidence-based as a phrase did show up three times but none were applicable to the topic. As to the term evidence, it was used n = 233 times. As the evidence relates to cognitive enhancements, one article states that the increased use of cosmetic neurology was presaged for a while [233]. Seven articles state that there was a lack of evidence; one article mentioned evidence of an underground market; two stated that there was evidence of increased acceptance, and four that there was evidence that cognitive enhancements are real. 

#### 3.4.3. Human Enhancement

As to assessment of human enhancement, the term shows up 138 times with various issues seen in need of being assessed, however technology assessment was only mentioned in one article stating the need for technology assessment, and other methods of analysis and reﬂection on the interface between technology and society [230]; impact assessment or needs assessment were not mentioned at all.

Evidence based as a phrase did not show up once, the term evidence showed up 87 times and was used to highlight that evidence is needed, that human enhancement technologies “are valued universally by the sorts of beings who will invest in developing them” [106,108], that there is no evidence that ‘more science education’ leads to increase in acceptance of a technology [108], and that there is evidence of ability trade-offs linked to cognitive enhancers [103]. One article looked at the role of physicians and contended that “evidence of the patient’s desire for the script is not enough to prescribe something” [179]. One article put forward the view that presuppositions of the value-neutrality of risk evaluations or ethics in the assessment of technological innovations are questionable and that a more complex analysis of human enhancement technologies is needed [105].

#### 3.4.4. Brain Machine Interface

With any new technology that has application in healthcare, and in other fields, a technology assessment is helpful (and often necessary) to determine the harms and benefits, costs, and user perspectives. However, there have been no published technology assessments performed with BMI technology. Only one article mentioned technology assessment, noting the relationship between technology development and technology assessment [182], although this was not linked to health technology assessment or any other kind of assessment. 

Related to assessment is the importance of evidence-based practice, and there were only two articles in our sample, which discussed evidence-based practice around BMI use. One paper discussed the dangers of using BMI without sufficient scientific evidence of effect and risks/benefits, and the importance of a clear evidence base for BMI comparable to clinical trial registries to aid evidence-based practice and research [117]. The other paper mentioned Cochrane reviews as a source of evidence for evidence-based practice [183]. However, for the majority of our sample, evidence is not discussed in these terms, but is rather seen as the output of experiments and basic scientific research, to prove effectiveness of BMI, rather than to guide decision-making [129,182,234,235,236,237,238].

#### 3.4.5. Social Robotics

In terms of assessment, the topic is mentioned in the social robotics literature, but as with the topic of policy, this is done only in passing. Two articles mention the need for assessment of robots used in gerontology to assess impact on outcomes [133,137]. However, such assessment is not the focus of this work, and there have currently been no published health technology assessments of robots used in healthcare.

#### 3.4.6. Discussion

Although we found various opinions on what should be assessed and various example of what has been assessed, the various existing fields of technology assessment as well as the fields of health needs, health impact and social impact assessment were absent from the academic literature we investigated even in the sense that one would find remarks as to the need to perform the various forms of assessment. As for one (parliamentary technology assessment), one might say that the results of this form of assessment are published in the form of reports and not academic papers. However searching Google or Bing, to just name two search engines, should reveal the extent to which reports on the various topics we covered are present. We found one report linked to parliamentary technology assessment [28] that covered various forms of human enhancement. To stay with human enhancement if we search Google or Bing for the term “technology assessment” and “human enhancement” we find reports from various technology assessment sources, such as from Switzerland, Germany, and the Netherlands; however, the existence of reports are by no means extensive. As to participatory technology assessment, one endeavour was found under that label [108]. As to the areas of health needs assessment and health impact assessment, there were very few to no hits in Google or Bing or our academic articles or Google Scholar, and the few hits obtained did not engage with the topics covered in this study in a substantial manner. This is troubling as the various therapeutic enhancement-linked products and discourses can be seen to have an impact on various health and health care fields.

However, what is striking is again the drop in findings if the health term is attached to it. Under the banner of health technology assessment none of the topics were assessed with the exception of [9] which covered some forms of enhancements. Given that fields such as health technology assessment have a horizon scanning component [239,240,241], that we see the European Commission being interested in human enhancement in a foresight manner [28], and given the many hits one obtains in Google and Google Scholar and in various academic databases on human enhancement and neuro/cognitive enhancements, and various therapeutic devices that can enable enhancements in the future, it is puzzling that none of the assessments fields investigated have a substantial body of work to show on the topic so far. Given the paradigm shift-enabling potential of human enhancement for the health field we submit that action is required here. The involvement of the different assessment fields might be one tool to trigger the generation of usable evidence in regards to numerous claims. Numerous academic papers around neuro and cognitive enhancement highlight the lack of decent evidence for the anecdotal claims of efficacy of enhancement drugs. Although the term evidence is appearing quite a bit, it is again curious that the health discourse-related term “evidence-based” is nearly invisible.

Finally the involvement of participatory technology assessment and social impact assessment might trigger a change in which social groups with which imagery are involved. In the case of our study we only looked at the imagery of disabled people, which to the greatest extend was medical with the focus on fixing the defect the disabled or impaired person experiences. What was totally missing was the presence of a disability studies lens, which sees the problem of disabled people originating from the physical and social environment [242,243,244,245,246,247,248,249,250,251,252,253]. Indeed Van Der Horst voiced the opinion that the 1992 guidelines of how to perform Social Impact Assessment are “more easily implemented in a social context that is familiar, and in a culture of governance that aims to be more inclusive and seeks to ensure the participation of all groups in the policy process, including weaker or more vulnerable groups in society such as the poor, women and children, people with disabilities, indigenous people, or other minorities” [254]. However as to disabled people, this has to include disabled people who do not want to get fixed.

## 4. Conclusions

We submit that all the science and technology products and discourses covered in this paper will impact the delivery of healthcare and the quality indicators of health, healthcare and health systems, such as the ones used in Canada [148,149], and already raise various health and healthcare policy issues. As such, it is troubling that health and healthcare policy is not more extensively covered within the discourses investigated in this study. Although some policy issues are raised they are by no means extensive. Furthermore, they are often raised by ethicists but not by health policy or healthcare policy scholars. Equally troubling is the issue that various assessment fields seem not to have engaged with the areas covered in this paper yet as have not the discourses around health consumerism. Finally it is troubling that disability is covered mostly from a medical angle, ignoring the broader issues of social determinants of health and how the discourses and products could impact in positive and negative way the social situation of disabled people. In general we submit there is a need for broadening the engagement with health and healthcare issues and the participation of a broader group of players in order for the discourses to be able to understand and deal with therapeutic enhancements and therapeutic enhancement enabling products.

## References

[1] Brand A., Brand H., den Baumen T.S. (2008). The impact of genetics and genomics on public health. Eur. J. Hum. Genet..

[2] Rickerby D.G. (2006). Societal and policy aspects of the introduction of nanotechnology in healthcare. Int. J. Healthc. Technol. Manag..

[3] Feeny D., Guyatt G., Tugwell P. (1986). Health Care Technology: Effectiveness, Efficiency, and Public Policy.

[4] Singer P. (1987). A report from Australia: Which babies are too expensive to treat?. Bioethics.

[5] Watts J.L., Blanchard R., Guyatt G., Miller D., Singer P., Haynes R.B., van Loon R. (1992). Technology in medicine—Ethics, politics and reality. Ann. R. Coll. Physicians Surg. Can..

[6] Singer P.A., Martin D.K., Giacomini M., Purdy L. (2000). Priority setting for new technologies in medicine: Qualitative case study. BMJ.

[7] Bates D.W., Bitton A. (2010). The future Of health information technology in the patient-centered medical home. Health Aff..

[8] Sorensen L. (2012). Health information technology and the transformation of health care in the 21st century. DNP Education, Practice, and Policy: Redesigning Advanced Practice Roles for the 21st Century.

[9] Wolbring G. (2005). HTA Initiative #23 The Triangle of Enhancement Medicine, Disabled People, and the Concept of Health: A New Challenge for HTA, Health Research, and Health Policy.

[10] Zettler P.J. (2009). Is it cheating to use cheetahs: The implications of technologically innovative prostheses for sports values and rules. Boston Univ. Int. Law J..

[11] Wolbring G. (2008). Oscar Pistorius and the future nature of Olympic, Paralympic and other sports. SCRIPTed J. Law Technol. Soc..

[12] International Paralympic Committee IPC Position Statement on IAAF’s Commissioned Research on Oscar Pistorius. http://www.paralympic.org/Media_Centre/News/General_News/2008_01_14_a.html/.

[13] Swartz L., Watermeyer B. (2008). Cyborg anxiety: Oscar Pistorius and the boundaries of what it means to be human. Disabil. Soc..

[14] Austin R., Ball P., Belcher A., Bensimon D., Chu S., Dekker C., Dyson F., Endy D., Fraser S., Glass J. The Ilulissat Statement Synthesizing the Future a Vision for the Convergence of Synthetic Biology and Nanotechnology. Kavli Futures Symposium ‘The Merging of Bio and Nano: Towards Cyborg Cells’. http://www.research.cornell.edu/KIC/images/pdfs/ilulissat_statement.pdf.

[15] Wolbring G. Artificial Hippocampus, the Borg Hive Mind, and Other Neurological Endeavors. http://www.innovationwatch.com/choiceisyours/choiceisyours.2006.11.15.htm/.

[16] Blakeslee S. (2008). Monkey’s Thoughts Propel Robot, a Step That May Help Humans. The New York Times.

[17] Corp A. Ambient Corp. http://www.theaudeo.com/tech.html/.

[18] Aylett R.S., Castellano G., Raducanu B., Paiva A., Hanheide M. Long-term Socially Perceptive and Interactive Robot Companions: Challenges and Future Perspectives. Proceedings of the 2011 ACM International Conference on Multimodal Interaction, ICMI ’11.

[19] Corley G. Intelligent OrthoSensor Devices Provide Real-Time Reporting on the Condition of your Orthopaedic Implant. http://medgadget.com/2011/07/intelligent-orthosensor-devices-provide-real-time-reporting-on-the-condition-of-your-orthopaedic-implant.html/.

[20] Partridge B.J., Bell S.K., Lucke J.C., Yeates S., Hall W.D. (2011). Smart drugs as common as coffee media hype about neuroenhancement. PLoS One.

[21] Schanker B.D. (2011). Neuroenhancement in a medicated generation: Overlooked uses of cognitive stimulants. AJOB Neurosci..

[22] Farah M.J. (2011). Overcorrecting the neuroenhancement discussion. Addiction.

[23] Wolbring G. (2011). Hearing beyond the normal enabled by therapeutic devices: The role of the recipient and the hearing profession. Neuroethics.

[24] Freitas R., Freitas R. (2003). 1.2.2 Volitional normative model of disease. Nanomedicine.

[25] Wolbring G., Lee Kleinmann D., Delborne J., Cloud-Hansen K., Handelsman J. (2010). Nanotechnology and the transhumanization of health, medicine, and rehabilitation. Controversies in Science & Technology.

[26] Wolbring G. (2006). Three challenges to the Ottawa spirit of health promotion, trends in global health, and disabled people. Can. J. Public Health.

[27] Wolbring G. (2012). Ethical theories and discourses through an ability expectations and ableism lens: The case of enhancement and global regulation. Asian Bioeth. Rev..

[28] Coenen C., Schuijff M., Smits M., Klaassen P., Hennen L., Rader M., Wolbring G. (2009). Human Enhancement Study.

[29] Jacob R., McGregor M. (1997). Assessing the impact of health technology assessment. Int. J. Technol. Assess. Health Care.

[30] Liberati A., Sheldon T.A., Banta H.D. (1997). EUR-ASSESS Project Subgroup report on Methodology. Methodological guidance for the conduct of health technology assessment. Int. J. Technol. Assess. Health Care.

[31] Drummond M., Weatherly H. (2000). Implementing the findings of health technology assessments. Int. J. Technol. Assess. Health Care.

[32] Lehoux P., Blume S. (2000). Technology assessment and the sociopolitics of health technologies. J. Health Polit. Policy Law.

[33] Rosenkoetter N., Vondeling H., Blancquaert I., Mekel O.C.L., Kristensen F.B., Brand A. (2011). The contribution of health technology assessment, health needs assessment, and health impact assessment to the assessment and translation of technologies in the field of public health genomics. Public Health Genomics.

[34] Bayoumi A.M., Krahn M. (2012). The future of health technology assessment. Med. Decis. Mak..

[35] Penney J.K. (2012). Qualitative evidence, knowledge translation, and policy-making, with reference to health technology assessment. Dalhous. J. Interdiscip. Manag..

[36] Haigh F., Harris P., Haigh N. (2012). Health impact assessment research and practice: A place for paradigm positioning?. Environ. Impact Assess. Rev..

[37] Mahoney M., Simpson S., Harris E., Aldrich R., Stewart-Williams J. (2004). Equity-Focused Health Impact Assessment Framework.

[38] Mahoney M., Morgan R.K. (2012). Health impact assessment in Australia and New Zealand: An exploration of methodological concerns. Promot. Educ..

[39] Hebert K.A., Wendel A.M., Kennedy S.K., Dannenberg A.L. (2012). Health impact assessment: A comparison of 45 local, national, and international guidelines. Environ. Impact Assess. Rev..

[40] Manitoba G.O. (2005). Community Health Needs Assessment Guidelines.

[41] Klute G.K., Kantor C., Darrouzet C., Wild H., Wilkinson S., Iveljic S., Creasey G. (2009). Lower-limb amputee needs assessment using multistakeholder focus-group approach. J. Rehabil. Res. Dev..

[42] Finsterbusch K. (1985). State of the art in social impact assessment. Environ. Behav..

[43] Becker H.A. (2001). Social impact assessment. Eur. J. Oper. Res..

[44] Vanclay F. (2004). The triple bottom line and impact assessment: How do TBL, EIA, SIA, SEA and EMS relate to each other?. J. Environ. Assess. Policy Manag..

[45] Hennen L. (2001). TA in Biomedicine and Healthcare—From clinical evaluation to policy consulting. TA-Datenbank Nachr..

[46] Delvenne P. Parliamentary Technology Assessment Performing Reflexivity: An Overview of TAB and STOA. Proceedings of the ITAS Kolloquium, Karlsruhe Institute of Technology.

[47] Weber K.M., Harper J.C., Könnölä T., Barceló V.C. (2012). Coping with a fast-changing world: Towards new systems of future-oriented technology analysis. Sci. Public Policy.

[48] Europta, EUROPTA Project (2002). European Participatory Technology Assessment. Participatory Methods in Technology Assessment and Technology Decision-Making.

[49] Brown N., Einsiedel E., Lundin S. (2010). Practicing Public Engagement in Controversial Science and Technology.

[50] Powell M., Kleinman D.L. (2008). Building citizen capacities for participation in nanotechnology decision-making: The democratic virtues of the consensus conference model. Public Underst. Sci..

[51] Cene C.W., Peek M.E., Jacobs E., Horowitz C.R. (2010). Community-based teaching about health disparities: Combining education, scholarship, and community service. J. Gen. Intern. Med..

[52] Morgan L.M. (2001). Community participation in health: Perpetual allure, persistent challenge. Health Policy Plan..

[53] Blackstock K.L., Kelly G.J., Horsey B.L. (2007). Developing and applying a framework to evaluate participatory research for sustainability. Ecol. Econ..

[54] Thorpe C., Gregory J. (2010). Producing the post-Fordist public: The political economy of public engagement with science. Sci. Cult..

[55] Merritt T. (2009). What can technology reviews contribute to participatory medicine?. J. Particip. Med..

[56] Levin-Zamir D., Peterburg Y. (2001). Health literacy in health systems: Perspectives on patient self-management in Israel. Health Promot. Int..

[57] Silber D. (2009). Medicine 2.0: The stakes of participatory medicine. Presse Med..

[58] Disch J. (2009). Participatory health care: Perspective from a nurse leader. J. Particip. Med..

[59] Lundberg G.D. (2009). Why healthcare professionals should practice participatory medicine: Perspective of a long-time medical editor. J. Particip. Med..

[60] Abelson J., Giacomini M., Lehoux P., Gauvin F.-P. (2007). Bringing “the public” into health technology assessment and coverage policy decisions: From principles to practice. Health Policy.

[61] Wolbring G. (2012). Therapeutic enhancements and the view of rehabilitation educators. Dilemata Int. J. Appl. Ethics.

[62] Taylor S., Shoultz B., Walker P. Disability Studies: Information and Resources. http://thechp.syr.edu/Disability_studies_2003_current.html/.

[63] Society for Disability Studies Guidelines for Disability Studies Programs. http://disstudies.org/guidelines-for-disability-studies-programs/.

[64] Conrad M.A., Fernald L.D. (1974). Nurses in Participatory Medicine—Hidden Curriculum of Acquiescence. Pa J. Physician’s Assoc..

[65] Kabat-Zinn J. (2000). Participatory medicine. J. Eur. Acad. Dermatol. Venereol..

[66] Snyderman R., Yoediono Z. (2008). Perspective: Prospective health care and the role of academic medicine: Lead, follow, or get out of the way. Acad. Med..

[67] Shen B. (2009). Bio-Socio-Technical Underpinnings of Participatory Medicine. J. Particip. Med..

[68] Swan M. (2012). Health 2050: The realization of personalized medicine through crowdsourcing, the quantified self, and the participatory biocitizen. J. Pers. Med..

[69] Ochocka J., Janzen R., Nelson G. (2002). Sharing power and knowledge: Professional and mental health consumer/survivor researchers working together in a participatory action research project. Psychiatr. Rehabil. J..

[70] Nelson G., Ochocka J., Janzen R., Trainor J. (2006). A longitudinal study of mental health consumer/survivor initiatives: Part 1—Literature review and overview of the study. J. Community Psychol..

[71] Nelson G., Janzen R., Trainor J., Ochocka J. (2008). Putting values into practice: Public policy and the future of mental health consumer-run organizations. Am. J. Commun. Psychol..

[72] Dunston R., Lee A., Boud D., Brodie P., Chiarella M. (2009). Co-production and health system reform—From re-imagining to re-making. Aust. J. Public Adm..

[73] Yergens D.R.J., Doig C.J. KSv2: Application for Enhancing Scoping and Systematic Reviews. Proceedings of American Medical Informatics Association (AMIA) 2012 Annual Symposium.

[74] Koenig T. Routinizing Frame Analysis through the Use of CAQDAS. Proceedings of the RC33 Sixth International Conference on Social Science Methodology.

[75] MacMillan K. (2005). More than just Coding? Evaluating CAQDAS in a Discourse Analysis of News Texts. Forum Qual. Soc. Res..

[76] Gini A., Rossi J., Giordano J. (2010). Considering enhancement (and/or treatment): On the need to regard contingency and develop dialectic evaluation—A commentary on Singh and Kelleher. AJOB Neurosci..

[77] Ravelingien A., Braeckman J., Crevits L., de Ridder D., Mortier E. (2009). ‘Cosmetic Neurology’ and the Moral Complicity Argument. Neuroethics.

[78] Singh I., Kelleher K.J. (2010). Neuroenhancement in young people: Proposal for research, policy, and clinical management. AJOB Neurosci..

[79] Benanti P. (2010). Neuroenhancement in young people: Cultural need or medical technology?. AJOB Neurosci..

[80] Bublitz J.C., Merkel R. (2009). Autonomy and authenticity of enhanced personality traits. Bioethics.

[81] Cooze J., Gillam L. (2010). Democratizing “Psychotropic Neuroenhancement”. AJOB Neurosci..

[82] Lev O. (2010). Should children have equal access to neuroenhancements?. AJOB Neurosci..

[83] Sahakian B.J., Morein-Zamir S. (2011). Neuroethical issues in cognitive enhancement. J. Psychopharmacol..

[84] Repantis D., Laisney O., Heuser I. (2010). Acetylcholinesterase inhibitors and memantine for neuroenhancement in healthy individuals: A systematic review. Pharmacol. Res..

[85] Larriviere D., Williams M.A., Rizzo M., Bonnie R.J. (2009). Responding to requests from adult patients for neuroenhancements. Neurology.

[86] Synofzik M. (2009). Ethically justified, clinically applicable criteria for physician decision-making in psychopharmacological enhancement. Neuroethics.

[87] Hall W. (2004). Feeling “better than well”: Can our experiences with psychoactive drugs help us to meet the challenges of novel neuroenhancement methods?. EMBO Reports.

[88] Hildt E. (2011). Neuroenhancement Bubble?—Neuroenhancement Wave!. AJOB Neurosci..

[89] Schleim S. (2010). Second thoughts on the prevalence of enhancement. BioSocieties.

[90] Bostrom N., Ord T. (2006). The Reversal Test: Eliminating Status Quo Bias in Applied Ethics*. Ethics.

[91] Lucke J.C. (2012). Empirical research on attitudes toward cognitive enhancement is essential to inform policy and practice guidelines. AJOB Prim. Res..

[92] Outram S.M., Racine E. (2011). Developing public health approaches to cognitive enhancement: An analysis of current reports. Public Health Ethics.

[93] Appel J.M. (2008). When the boss turns pusher: A proposal for employee protections in the age of cosmetic neurology. J. Med. Ethics.

[94] Hogle L.F. (2005). Enhancement technologies and the body. Annu. Rev. Anthropol..

[95] Howell R.D. (1990). Technology and change in special education: An interactional perspective. Theory Pract..

[96] McNamee M., Edwards S. (2006). Transhumanism, medical technology and slippery slopes. J. Med. Ethics.

[97] Bostrom N., Roache R. (2010). Smart Policy: Cognitive Enhancement and the Public Interest. Contemp. Read. Law Soc. Justice.

[98] Drabiak Syed K. (2011). Reining in the pharmacological enhancement train: We should remain vigilant about regulatory standards for prescribing controlled substances. J. Law Med. Ethics.

[99] Hagger L., Johnson G.H. (2011). “Super kids”: Regulating the use of cognitive and psychological enhancement in children. Law Innov. Technol..

[100] Bell S.K., Lucke J.C., Hall W.D. (2012). Lessons for enhancement from the history of cocaine and amphetamine use. AJOB Neurosci..

[101] Whitehouse P.J., Juengst E., Mehlman M., Murray T.H. (1997). Enhancing cognition in the intellectually intact. Hastings Cent. Rep..

[102] Fenton E. (2010). The perils of failing to enhance: A response to Persson and Savulescu. J. Med. Ethics.

[103] De Jongh R., Bolt I., Schermer M., Olivier B. (2008). Botox for the brain: Enhancement of cognition, mood and pro-social behavior and blunting of unwanted memories. Neurosci. Biobehav. Rev..

[104] Kin C.A. (1996). Coming soon to the “genetic supermarket” near you. Stanford Law Rev..

[105] De Melo-Martín I. (2010). Defending human enhancement technologies: Unveiling normativity. J. Med. Ethics.

[106] Bradshaw H.G., ter Meulen R. (2010). A transhumanist fault line around disability: Morphological freedom and the obligation to enhance. J. Med. Philos..

[107] Wolbring G. (2009). “Therapeutic”, enhancement enabling, assistive devices and the UN Convention on the Rights of Persons with Disabilities: A missing lens in the enhancement regulation discourse. J. Int. Biotechnol. Law.

[108] Cobb M.D. (2011). Creating informed public opinion: Citizen deliberation about nanotechnologies for human enhancements. J. Nanopart. Res..

[109] Cullen D. (2007). Back to the future: Eugenics-a bibliographic essay. Public Hist..

[110] Forlini C., Racine E. (2011). Considering the Causes and Implications of Ambivalence in Using Medicine for Enhancement. The American Journal of Bioethics.

[111] Juengst E.T., Binstock R.H., Mehlman M., Post S.G., Whitehouse P. (2003). Biogerontology, anti-aging medicine and the challenges of human enhancement. Hastings Cent. Rep..

[112] Weldon S., Laycock D. (2009). Public opinion and biotechnological innovation. Policy Soc..

[113] Clough B.A., Casey L.M. (2011). Technological adjuncts to enhance current psychotherapy practices: A review. Clin. Psychol. Rev..

[114] Mason S.G., Bashashati A., Fatourechi M., Navarro K.F., Birch G.E. (2007). A comprehensive survey of brain interface technology designs. Ann. Biomed. Eng..

[115] Jackson M.M.M., Mason S.G., Birch G.E. (2006). Analyzing trends in brain interface technology: A method to compare studies. Ann. Biomed. Eng..

[116] Elder J.B., Hoh D.J., Oh B.C., Heller A.C., Liu C.Y., Apuzzo M.L.J. (2008). The future of cerebral surgery: A kaleidoscope of opportunities. Neurosurgery.

[117] Clausen J. (2011). Conceptual and ethical issues with brain-hardware interfaces. Curr. Opin. Psychiatry.

[118] Neven L. (2010). But obviously not for me: Robots, laboratories and the defiant identity of elder test users. Sociol. Health Illn..

[119] McCullagh P.J., Ware M., Mulvenna M., Lightbody G., Nugent C.D., McAllister H.G., Thomson E., Martin S., Mathews S., Todd D. (2010). Can brain computer interfaces become practical assistive devices in the community?. Stud. Health Technol. Inform..

[120] Isa T., Fetz E.E., Müller K.R. (2009). Editorial: Recent advances in brain-machine interfaces. Neural Netw..

[121] Menon C., de Negueruela C., Milláne J.R., Tonet O., Carpi F., Broschart M., Ferrez P., Buttfield A., Tecchio F., Sepulveda F. (2009). Prospects of brain-machine interfaces for space system control. Acta Astronaut..

[122] Mussa-Ivaldi F.A., Miller L.E. (2003). Brain-machine interfaces: Computational demands and clinical needs meet basic neuroscience. Trends Neurosci..

[123] Martin A.R., Sankar T., Lipsman N., Lozano A.M. (2012). Brain-machine interfaces for motor control: A guide for neuroscience clinicians. Can. J. Neurol. Sci..

[124] Hatsopoulos N.G., Donoghue J.P. (2009). The science of neural interface systems. Annu. Rev. Neurosci..

[125] Allison B. The I of BCIs: Next Generation Interfaces for Brain Computer Interface Systems That Adapt to Individual Users. Proceedings of the 13th International Conference of Human-Computer Interaction.

[126] Patil S.A. Brain Gate as an Assistive and Solution Providing Technology for Disabled People. Proceedings of the 13th International Conference on Biomedical Engineering.

[127] Bostrom N., Sandberg A. (2009). Cognitive enhancement: Methods, ethics, regulatory challenges. Sci. Eng. Ethics.

[128] Demetriades A.K., Demetriades C.K., Watts C., Ashkan K. (2010). Brain-machine interface: The challenge of neuroethics. Surgeon.

[129] Birbaumer N., Cohen L.G. (2007). Brain-computer interfaces: Communication and restoration of movement in paralysis. J. Physiol..

[130] Yumakulov S., Yergens D., Wolbring G. Imagery of People with Disabilities within Social Robotics Research. Proceedings of the 4th International Conference of Social Robotics, ICSR 2012.

[131] Sugiyama O., Shinozawa K., Akimoto T., Hagita N. Case Study of a Multi-robot Healthcare System: Effects of Docking and Metaphor on Persuasion. Proceedings of the 2nd International Conference on Social Robotics, ICSR 2010.

[132] Broadbent E., Lee Y.I., Stafford R.Q., Kuo I.H., MacDonald B.A. (2011). Mental schemas of robots as more human-like are associated with higher blood pressure and negative emotions in a human-robot interaction. Int. J. Soc. Robot..

[133] Gelderblom G.J., Bemelmans R., Spierts N., Jonker P., de Witte L. Development of PARO Interventions for Dementia Patients in Dutch Psycho-geriatric Care. Proceedings of the 2nd International Conference on Social Robotics, ICSR 2010.

[134] Kuo I.H., Jayawardena C., Broadbent E., MacDonald B.A. (2011). Multidisciplinary design approach for implementation of interactive services: Communication initiation and user identification for healthcare service robots. Int. J. Soc. Robot..

[135] Tamagawa R., Watson C.I., Kuo I.H., MacDonald B.A., Broadbent E. (2011). The effects of synthesized voice accents on user perceptions of robots. Int. J. Soc. Robot..

[136] Wasen K. (2010). Replacement of highly educated surgical assistants by robot technology in working life: Paradigm shift in the service sector. Int. J. Soc. Robot..

[137] Broadbent E., Stafford R., MacDonald B. (2009). Acceptance of healthcare robots for the older population: Review and future directions. Int. J. Soc. Robot..

[138] Moon A.J., Danielson P., van der Loos H.F.M. (2012). Survey-based discussions on morally contentious applications of interactive robotics. Int. J. Soc. Robot..

[139] Salvini P., Laschi C., Dario P. (2010). Design for acceptability: Improving robots’ coexistence in human society. Int. J. Soc. Robot..

[140] Lin P., Abney K., Bekey G. (2011). Robot Ethics: Mapping the Issues for a Mechanized World. Artif. Intell..

[141] Sparrow R., Sparrow L. (2006). In the hands of machines? The future of aged care. Minds Mach..

[142] Levy D. (2009). The ethical treatment of artificially conscious robots. Int. J. Soc. Robot..

[143] Weng Y.H. (2010). Beyond robot ethics: On a legislative consortium for social robotics. Adv. Robot..

[144] Wiegel V., van den Berg J. (2009). Combining moral theory, modal logic and mas to create well-behaving artificial agents. Int. J. Soc. Robot..

[145] Coeckelbergh M. (2009). Personal robots, appearance, and human good: A methodological reflection on roboethics. Int. J. Soc. Robot..

[146] Sabanovic S. (2010). It takes a village to construct a robot: A socially situated perspective on the ethics of robot design. Interact. Stud..

[147] Shaw-Garlock G. (2009). Looking forward to sociable robots. Int. J. Soc. Robot..

[148] Health Quality Council of Alberta (2005). Alberta Quality Matrix for Health of the Health Quality Council of Alberta. http://www.hqca.ca/assets/pdf/Matrix%20.pdf.

[149] Canadian Institute of Health Information Health Indicators 2010. https://secure.cihi.ca/estore/productFamily.htm?pf=PFC1435/.

[150] Williams A.E. Good, Better, Best: The Human Quest for Enhancement Summary Report of an Invitational Workshop Convened by the Scientific Freedom, Responsibility and Law Program American Association for the Advancement of Science. http://www.aaas.org/spp/sfrl/projects/human_enhancement/pdfs/HESummaryReport.pdf.

[151] Daniels N., Saloner B., Gelpi A.H. (2009). Access, cost, and financing: Achieving an ethical health reform. Health Aff..

[152] Jecker N.S. (2008). A broader view of justice. Am. J. Bioeth..

[153] Kamm F.M. (1987). The choice between people: ‘common sense’ morality, and doctors. Bioethics.

[154] Marchand S., Wikler D., Landesman B. (1998). Class, health, and justice. Milbank Q..

[155] Sherwin S., Baylis F. (2003). The feminist health care ethics consultant as architect and advocate. Public Aff.Q..

[156] Caplan A.L., Callahan D., Haas J. (1987). Ethical and policy issues in rehabilitation medicine. Hastings Cent.Rep..

[157] Singer P. (1988). Setting limits: Medical goals in an aging society, by Daniel Callahan. Bioethics.

[158] Bayer R., Callahan D., Caplan A.L., Jennings B. (1988). Toward justice in health care. Am. J. Public Health.

[159] Olsen J.A. (1997). Theories of justice and their implications for priority setting in health care. J. Health Econ..

[160] Cookson R., Dolan P. (2000). Principles of justice in health care rationing. J. Med. Ethics.

[161] Gakidou E.E., Murray C.J., Frenk J. (2000). Defining and measuring health inequality: An approach based on the distribution of health expectancy. Bull. World Health Organ..

[162] Savulescu J. (1999). Desire-based and value-based normative reasons. Bioethics.

[163] Rutten F.F.H., Bonsel G.J. (1992). High cost technology in health care: A benefit or a burden?. Soc. Sci. Med..

[164] Dargie C. (2000). Policy Futures for UK Health 2000 Report Part 2 Analysing Issues for Health in 2015 Rising Public Expectations.

[165] Murphy N.J. (2005). Citizen deliberation in setting health-care priorities. Health Expect..

[166] Kashefi E., Mort M. (2004). Grounded citizens’ juries: A tool for health activism?. Health Expect..

[167] Guttman N., Shalev C., Kaplan G., Abulafia A., Bin-Nun G., Goffer R., Ben-Moshe R., Tal O., Shani M., Lev B. (2008). What should be given a priority-costly medications for relatively few people or inexpensive ones for many? The Health Parliament public consultation initiative in Israel. Health Expect..

[168] Menon D. Which Kind of Health Technologies Should We Assess and Why? A Citizens’ Jury Delivered its Verdict. http://www.hinnovic.org/which-kind-of-health-technologies-should-we-assess-and-why/.

[169] Wolbring G., Wilsdon J.M.P. (2006). The Unenhanced Underclass. Better Humans? The Politics of Human Enhancement.

[170] Mendelsohn D., Lipsman N., Bernstein M. (2010). Neurosurgeons’ perspectives on psychosurgery and neuroenhancement: A qualitative study at one center: Clinical article. J. Neurosurg..

[171] Choudhury S., Nagel S.K., Slaby J. (2009). Critical neuroscience: Linking neuroscience and society through critical practice. BioSocieties.

[172] Palmour N., Racine E. (2011). Direct-to-consumer marketing of dietary supplements for dementia: An example of unhealthy commerce of neuroscience. AJOB Neurosci..

[173] Hall W. (2004). Feeling “better than well”. EMBO Rep..

[174] Larriviere D., Williams M.A. (2010). Neuroenhancement: Wisdom of the masses orö false phronesisö?. Clin. Pharmacol. Ther..

[175] Turner D.C., Sahakian B.J. (2006). Ethical questions in functional neuroimaging and cognitive enhancement. Poiesis Prax..

[176] Banjo O.C., Nadler R., Reiner P.B. (2010). Physician attitudes towards pharmacological cognitive enhancement: Safety concerns are paramount. PLoS One.

[177] Coveney C., Gabe J., Williams S. (2011). The sociology of cognitive enhancement: Medicalisation and beyond. Health Sociol. Rev..

[178] Outram S.M. (2010). The use of methylphenidate among students: The future of enhancement?. J. Med. Ethics.

[179] Kirschner K.L., Dreger A., Wolbring G. (2010). Brave new world? Enhancement and rehabilitation medicine. PM&R.

[180] Leong C.C., Jarvis D., Howlett M., Migone A. (2011). Controversial science-based technology public attitude formation and regulation in comparative perspective: The state construction of policy alternatives in Asia. Technol. Soc..

[181] De Roubaix J. (2011). Beneficence, non-maleficence, distributive justice and respect for patient autonomy: Reconcilable ends in aesthetic surgery?. J. Plast. Reconstr. Aesthet. Surg..

[182] Mason S.G., Jackson M.M.M., Birch G.E. (2005). A general framework for characterizing studies of brain interface technology. Ann. Biomed. Eng..

[183] Packman A., Meredith G. (2011). Technology and the evolution of clinical methods for stuttering. J. Fluen. Disord..

[184] Breazeal C. (2009). Role of expressive behaviour for robots that learn from people. Philos. Trans. R. Soc. B Biol. Sci..

[185] Young J.E., Hawkins R., Sharlin E., Igarashi T. (2009). Toward acceptable domestic robots: Applying insights from social psychology. Int. J. Soc. Robot..

[186] De Ruyter B., Saini P., Markopoulos P., van Breemen A. (2005). Assessing the effects of building social intelligence in a robotic interface for the home. Interact. Comput..

[187] Seale C., Cavers D., Dixon-Woods M. (2006). Commodification of body parts: By medicine or by media?. Body Soc..

[188] Ungerson C. (1997). Social politics and the commodification of care. Soc. Polit..

[189] Stern M. (2003). Shiny, happy people: Body Worlds’ and the commodification of health. Radic. Philos..

[190] Negrin L. (2002). Cosmetic surgery and the eclipse of identity. Body Soc..

[191] Davis K. (1995). Reshaping the Female Body.

[192] Swami V., Chamorro-Premuzic T., Bridges S., Furnham A. (2009). Acceptance of cosmetic surgery: Personality and individual difference predictors. Body Image.

[193] Hacking I. (1998). Canguilhem amid the cyborgs. Econ. Soc..

[194] Leng K.W. (1996). On menopause and cyborgs: Or, towards a feminist cyborg politics of menopause. Body Soc..

[195] Haraway D. (1991). Simians, Cyborgs and Women: The Reinvention of Nature.

[196] Canguilhem G. (1952). La Connaissance de la vie: Machine et Organisme.

[197] Wiener N. (1965). Cybernetics or Control and Communication in the Animal and the Machine.

[198] Bostrom N. (2005). In defense of posthuman dignity. Bioethics.

[199] Coughlin J.F., Pope J.E., Leedle B.R. (2006). Old age, new technology, and future innovations in disease management and home health care. Home Health Care Manag. Pract..

[200] Frydman G.J. (2009). Patient-driven research: Rich opportunities and real risks. J. Particip. Med..

[201] Swan M. (2009). Emerging patient-driven health care models: an examination of health social networks, consumer personalized medicine and quantified self-tracking. Int. J. Environ. Res. Public Health.

[202] Dvorsky G. The Quantified Self: 6 Tools to Help You Get Started. http://ieet.org/index.php/IEET/more/dvorsky20101106/.

[203] Wolf G. The quantified self. http://www.ted.com/talks/gary_wolf_the_quantified_self.html/.

[204] Blaze Carlson K. The Quantified Self by the Numbers. The National Post. http://news.nationalpost.com/2010/10/02/the-quantified-self-by-the-numbers/.

[205] Bloss C.S., Ornowski L., Silver E., Cargill M., Vanier V., Schork N.J., Topol E.J. (2010). Consumer perceptions of direct-to-consumer personalized genomic risk assessments. Genet. Med..

[206] Guttmacher A.E., McGuire A.L., Ponder B., Stefánsson K. (2010). Personalized genomic information: Preparing for the future of genetic medicine. Nat. Rev. Genet..

[207] Kato K., Kano K., Shirai T. (2010). Science communication: Significance for genome-based personalized medicineûa view from the Asia-Pacific. Curr. Pharmacogenomics.

[208] Keller M.A., Gordon E.S., Stack C.B., Gharani N., Sill C.J., Schmidlen T.J., Joseph M., Pallies J., Gerry N.P., Christman M.F. (2010). Coriell Personalized Medicine Collaborative^©^: A prospective study of the utility of personalized medicine. Pers. Med..

[209] Conrad P. (1975). The discovery of hyperkinesis: Notes on the medicalization of deviant behavior. Soc. Probl..

[210] Illich I. (1975). The medicalization of life. J. Med. Ethics.

[211] Searight R.H., McLaren L.A. (1998). Attention-Deficit Hyperactivity Disorder: The medicalization of misbehavior. J. Clin. Psychol. Med. Settings.

[212] Malacrida C. (2004). Medicalization, ambivalence and social control: Mothers’ descriptions of educators and ADD/ADHD. Health (Lond.).

[213] Conrad P. (2007). The Medicalization of Society: On the Transformation of Human Conditions into Treatable Disorders.

[214] Suissa A.J. (2008). Addiction to cosmetic surgery: Representations and medicalization of the body. Int. J. Ment. Health Addict..

[215] Coveney C.M., Nerlich B., Martin P. (2009). Modafinil in the media: Metaphors, medicalisation and the body. Soc. Sci. Med..

[216] Moynihan R., Heath I., Henry D. (2002). Selling sickness: The pharmaceutical industry and disease mongering. BMJ.

[217] Polonijo A.N., Carpiano R.M. (2008). Representations of cosmetic surgery and emotional health in women’s magazines in Canada. Womens Health Issues.

[218] Brooks A. (2004). “Under the knife and proud of it:” * An analysis of the normalization of cosmetic surgery. Crit. Sociol..

[219] Scott C.M., Horne T., Thurston W.E. (2002). The Differential Impact of Health Care Privatization on Women in Alberta. Exposing Privatization: Women and Health Care Reform in Canada.

[220] Rosenthal M., Daniels N. (2006). Beyond competition: The normative implications of consumer-driven health plans. J. Health Polit. Policy Law.

[221] Mossialos E. (1997). Citizens’ views on health care systems in the 15 member states of the European Union. Health Econ..

[222] Buntin M.B., Damberg C., Haviland A., Kapur K., Lurie N., McDevitt R., Marquis M.S. (2006). Consumer-directed health care: Early evidence about effects on cost and quality. Health Aff..

[223] Carman K.L., Maurer M., Yegian J.M., Dardess P., McGee J., Evers M., Marlo K.O. (2010). Evidence that consumers are skeptical about evidence-based health care. Health Aff..

[224] Bechtel C., Ness D.L. (2010). If you build it, will they come? Designing truly patient-centered health care. Health Aff..

[225] Murray C.J., Kawabata K., Valentine N. (2001). People’s experience versus people's expectations. Health Aff. (Millwood).

[226] Nord E. (1989). The significance of contextual factors in valuing health states. Health Policy.

[227] Mossialos E., Thomson S.M. (2002). Voluntary health insurance in the European Union: A critical assessment. Int. J. Health Serv..

[228] Berger F., Gevers S., Siep L., Weltring K.M. (2008). Ethical, legal and social aspects of brain-implants using nano-scale materials and techniques. NanoEthics.

[229] Franke A.G., Bonertz C., Christmann M., Engeser S., Lieb K. (2012). Attitudes toward cognitive enhancement in users and nonusers of stimulants for cognitive enhancement: A pilot study. AJOB Prim. Res..

[230] Grunwald A. (2007). Converging technologies: Visions, increased contingencies of the conditio humana, and search for orientation. Futures.

[231] Repantis D., Schlattmann P., Laisney O., Heuser I. (2010). Modafinil and methylphenidate for neuroenhancement in healthy individuals: A systematic review. Pharmacol. Res..

[232] Sahakian B., Morein-Zamir S. (2007). Professor’s little helper. Nature.

[233] Cakic V. (2009). Smart drugs for cognitive enhancement: Ethical and pragmatic considerations in the era of cosmetic neurology. J. Med. Ethics.

[234] Birbaumer N., Murguialday A.R., Cohen L. (2008). Brain-computer interface in paralysis. Curr. Opin. Neurol..

[235] Wolpaw J.R., Birbaumer N., McFarland D.J., Pfurtscheller G., Vaughan T.M. (2002). Brain-computer interfaces for communication and control. Clin. Neurophysiol..

[236] Suminski A.J., Tkach D.C., Hatsopoulos N.G. (2009). Exploiting multiple sensory modalities in brain-machine interfaces. Neural Netw..

[237] Jerbi K., Vidal J.R., Mattout J., Maby E., Lecaignard F., Ossandon T., Hamamé C.M., Dalal S.S., Bouet R., Lachaux J.P. (2011). Inferring hand movement kinematics from MEG, EEG and intracranial EEG: From brain-machine interfaces to motor rehabilitation. IRBM.

[238] Serruya M.D., Kahana M.J. (2008). Techniques and devices to restore cognition. Behav. Brain Res..

[239] The National Horizon Scanning Centre UK, Horizon Scanning Centre, National Institute of Health Research NIHR Horizon Scanning Centre (NIHR HSC), UK. http://www.nice.org.uk/aboutnice/whatwedo/aboutmedicaltechnologies/innovationlandscape/productdevelopment/NationalHorizonScanningCentre.jsp/.

[240] Kolasa K., Kalo Z., Zah V., Dolezal T. (2012). Role of health technology assessment in the process of implementation of the EU Transparency Directive: Relevant experience from Central Eastern European countries. Expert Rev. Pharmacoecon. Outcomes Res..

[241] Walley T. (2012). Translating comparative effectiveness research into clinical practice: The UK experience. Drugs.

[242] Imrie R. (1996). Ableist geographies, disablist spaces: Towards a reconstruction of Golledge’s ‘Geography and the disabled’. Trans. Inst. Br. Geogr..

[243] Ayim M. (1997). Crimes against the Deaf: The Politics of Ableism. Can. J. Educ..

[244] Livingston K. (2000). When architecture disables: Teaching undergraduates to perceive ableism in the built environment. Teach. Sociol..

[245] Carlson L. (2001). Cognitive ableism and disability studies: Feminist reflections on the history of mental retardation. Hypatia.

[246] Hehir T. (2002). Eliminating ableism in education. Harv. Educ. Rev..

[247] Overboe J. (2007). Vitalism: Subjectivity Exceeding Racism, Sexism, and (Psychiatric) Ableism. Wagadu.

[248] Wolbring G. (2008). The politics of ableism. Development.

[249] Campbell F.K. (2008). Refusing Able (ness): A Preliminary Conversation about Ableism. M/C J..

[250] Wolbring G. (2012). Expanding ableism: Taking down the ghettoization of impact of disability studies scholars. Societies.

[251] Wolbring G., Ball N. (2012). Nanoscale science and technology and people with disabilities in Asia: An ability expectation analysis. NanoEthics.

[252] Wolbring G., Lightman A., Sarewitz D., Desser C. (2003). Confined to your legs. Living with the Genie.

[253] Wolbring G., Bainbridge W.S. (2003). Science and technology and the triple D (disease, disability, defect). Converging Technologies for Improving Human Performance: Nanotechnology, Biotechnology, Information Technology and Cognitive Science.

[254] Van der Horst D., Vermeylen S. (2011). Spatial scale and social impacts of biofuel production. Biomass Bioenergy.

